# Novel genetic alterations in liver cancer distinguish distinct clinical outcomes and combination immunotherapy responses

**DOI:** 10.3389/fphar.2024.1416295

**Published:** 2024-06-14

**Authors:** Yizhou Wang, Peipei Shang, Chang Xu, Wei Dong, Xiaofeng Zhang, Yong Xia, Chengjun Sui, Cheng Yang

**Affiliations:** ^1^ Department of Hepatic Surgery IV and Clinical Research Institute, Eastern Hepatobiliary Surgery Hospital, Third Affiliated Hospital of Naval Medical University, Shanghai, China; ^2^ Department of Medical Oncology, Eastern Hepatobiliary Surgery Hospital, Third Affiliated Hospital of Naval Medical University, Shanghai, China; ^3^ Department of General Surgery, Biliary Tract Disease Institute, Biliary Tract Disease Center, and Cancer Center of Zhongshan Hospital, Fudan University, Shanghai, China; ^4^ Department of Pathology, Eastern Hepatobiliary Surgery Hospital, Third Affiliated Hospital of Naval Medical University, Shanghai, China; ^5^ Department of Special Treatment, Eastern Hepatobiliary Surgery Hospital, Third Affiliated Hospital of Naval Medical University, Shanghai, China; ^6^ Department of Interventional Oncology, Renji Hospital, School of Medicine, Shanghai Jiao Tong University, Shanghai, China

**Keywords:** liver cancer, targeted sequencing, vascular invasion, overall survival, recurrence-free survival, immunotherapy, progression-free survival

## Abstract

**Introduction:** Genomic profiling has revolutionized therapeutic interventions and the clinical management of liver cancer. However, pathogenetic mechanisms, molecular determinants of recurrence, and predictive biomarkers for first-line treatment (anti-PD-(L)1 plus bevacizumab) in liver cancer remain incompletely understood.

**Materials and methods:** Targeted next-generation sequencing (tNGS) (a 603-cancer-gene panel) was applied for the genomic profiling of 232 hepatocellular carcinoma (HCC) and 22 intrahepatic cholangiocarcinoma (ICC) patients, among which 47 unresectable/metastatic HCC patients underwent anti-PD-1 plus bevacizumab therapy. Genomic alterations were estimated for their association with vascular invasion (VI), location of onset, recurrence, overall survival (OS), recurrence-free survival (RFS), and anti-PD-1 plus bevacizumab therapy response.

**Results:** The genomic landscape exhibited that the most commonly altered genes in HCC were *TP53*, *FAT3*, *PDE4DIP*, *KMT2C*, *FAT1*, and *MYO18A*, while *TP53*, *FAT1*, *FAT3*, *PDE4DIP*, *ROS1*, and *GALNT11* were frequently altered in ICC; notably, *KRAS* (18.18% vs. 1.29%) and *BAP1* (13.64% vs. 1.29%) alterations were significantly more prevalent in ICC. Comparison analysis demonstrated the distinct clinicopathological/genomic characterizations between Chinese and Western HCC cohorts. Genomic profiling of HCC underlying VI showed that *LDLR*, *MSH2*, *KDM5D*, *PDE3A*, and *FOXO1* were frequently altered in the VI group compared to patients without VIs. Compared to the right hepatic lobes of HCC patients, the left hepatic lobe of HCC patients had superior OS (median OS: 36.77 months vs. unreached, *p* < 0.05). By further comparison, Notch signaling pathway-related alterations were significantly prevalent among the right hepatic lobes of HCC patients. Of note, multivariate Cox regression analysis showed that altered *RB1*, *NOTCH3*, *MGA*, *SYNE1*, and *ZFHX3*, as independent prognostic factors, were significantly correlated with the OS of HCC patients. Furthermore, altered *LATS1* was abundantly enriched in the HCC-recurrent group, and impressively, it was independent of clinicopathological features in predicting RFS (median RFS of altered type vs. wild-type: 5.57 months vs. 22.47 months, *p* < 0.01). Regarding those treated HCC patients, TMB value, altered *PTPRZ1*, and cell cycle-related alterations were identified to be positively associated with the objective response rate (ORR), but *KMT2D* alterations were negatively correlated with ORR. In addition, altered *KMT2D* and cell cycle signaling were significantly associated with reduced and increased time to progression-free survival (PFS), respectively.

**Conclusion:** Comprehensive genomic profiling deciphered distinct molecular characterizations underlying VI, location of onset, recurrence, and survival time in liver cancer. The identification of novel genetic predictors of response to anti-PD-1 plus bevacizumab in HCC facilitated the development of an evidence-based approach to therapy.

## 1 Introduction

Hepatocellular carcinoma (HCC), as the major subtype of liver cancer, is one of the most common malignancies across the world (Vogel et al., 2022). Undoubtedly, HCC is also one of the most pandemic-diagnosed cancers in China, and the newest cancer statistics from the National Cancer Center of China manifested that the estimated incidence and mortality rates of HCC have increased to 28.12/10^5^ and 24.33/10^5^, respectively (Zheng et al., 2022). In clinical practice, viral infection (hepatitis B and C virus, namely, HBV and HCV) (Chen, 2018), alcohol consumption (Morgan et al., 2004), obesity (Obesity Independently Drives NASH and, 2018), environmental exposures (such as aflatoxin) (Yu and Yuan, 2004), and non-alcoholic fatty liver disease (Debes et al., 2020) have become the predominant etiological factors for HCC. Furthermore, it is generally thought that these etiological factors can lead to chronic cirrhosis highly associated with the development of HCC; however, comprehensive population-based studies reveal that it is still controversial whether cirrhosis is a predisposing factor in developing HCC (Garrido and Djouder, 2021). As is known, HCC is a type of heterogeneous malignancy (Schulze et al., 2016). Nowadays, molecular profiling provides a novel insight into the development and progression of HCC (Sia and Llovet, 2017), offers a pathomolecular approach, and identifies a variety of tumor subtypes (Paradis, 2021). Moreover, genomic characterization deciphers the relationship between molecular heterogeneity and therapeutic intervention/clinical outcome in HCC (Lin et al., 2017). In brief, the genomic characterization of HCC is instructive for the development of precision medicine and will be of substantial value in promoting clinical management.

Previously, several studies with medium-sized cohorts have explored the genomic characterizations of HCC from a wide range of genetic backgrounds. A prospective cohort study of 231 Korean patients with HCC (Ahn et al., 2014) discovered that *TP53*, *VCX*, *CTNNB1*, *AXIN1*, *SELPLG*, *RB1*, *ALB*, *RPS6KA3*, and *CDKN1B* were markedly altered, and genomic aberrations were enriched in the chromatin remodeling, cell cycle, and WNT, PI3K/RAS, and TP53 pathways. Interestingly, it was further found that *RB1* mutations and genomic aberrations in the cell cycle pathway were significantly correlated with disease-specific survival (DSS) and recurrence-free survival (RFS) in the Korean cohort, respectively. Subsequently, another Asian HCC cohort with 300 Japanese patients (Fujimoto et al., 2016) revealed that *TERT* promoter mutations were correlated with cigarette smoking, *LRP1B* mutations were correlated with alcohol consumption, and additionally, *BRD7* and *CTTNB1* mutations were correlated with HBV and HCV, respectively. In addition, Japanese HCC patients with *ARID2/PBRM1* or *TP53* mutations had worse disease-free survival (DFS); in contrast, patients with *MACROD2* mutations had better DFS. However, exome sequencing analysis from a French cohort (Schulze et al., 2015) revealed that alcohol-associated HCC patients had enriched mutations in *CTNNB1*, *TERT*, *CDKN2A*, *SMARCA2*, and *HGF*, while *TP53* mutations were frequently observed among HBV-related HCC patients. Notably, French HCC patients with *CDKN2A* mutations had significantly worse overall survival (OS) than those without *CDKN2A* mutations. Afterward, the analysis of the United States HCC cohort (Ally et al., 2017), also known as TCGA (The Cancer Genome Atlas) HCC cohort, displayed that frequent mutations were enriched in the *TERT* promotor region, *TP53*, and *CTNNB1*. Collectively, multiple studies have identified that there are common driver genes, such as *CTNNB1*, and genes responsible for telomere maintenance, p53 signaling, Wnt signaling, chromatin remodeling, cell cycle, and kinase signaling that are prevalently mutated in HCC (Rebouissou and Nault, 2020). In addition, the prospective genotyping of HCC recognized that oncogenic alterations in PI3K–mTOR signaling might predict the resistance of sorafenib in HCC, and an immune-excluded HCC subtype was characterized with WNT/CTNNB1 mutations, which could predict resistance to immune checkpoint protein inhibitors, serving as an immunotherapy biomarker (Pinyol et al., 2019a; Harding et al., 2019).

Recently, a comprehensive retrospective study of 1,349 HCC patients from 5 cohorts revealed that a lot of genomic events, consisting of driver genes, tumor mutational burden (TMB), mutational signature, and even copy number alteration, segregated distinctively across HCC patients from different genetic backgrounds, therefore highlighting the importance of differential cancer biology and clinical management in HCC across a wide range of genetic backgrounds (Kaya et al., 2022). Currently, there are a few studies with small cohorts investigating the genomic characterization of Chinese HCC patients (Ling et al., 2018; Gao et al., 2019; Wang et al., 2019; Yang et al., 2023) and finding that *TP53*, *TERT*, and *CTNNB1* were the most frequently altered mutations in Chinese HCC. Despite these, there still existed a considerable proportion of Chinese HCC patients not harboring any known driver mutations, indicating that the genomic characterization of Chinese HCC patients remained to be fully elucidated. More importantly, an in-depth investigation of the genomic landscape could help understand the pathogenetic mechanism of HCC, which would leverage great advances in promoting precision medicine and clinical management. In this study, a total of 232 HCC patients along with 22 patients with intrahepatic cholangiocarcinoma (ICC) were employed, and their tumor tissues were enrolled in targeted next-generation sequencing (tNGS) to depict the landscape of genomic alterations in Chinese HCC/ICC patients. Meanwhile, we analyzed the association of genomic mutations with clinicopathological features, prognosis, and the response of anti-PD-1 plus bevacizumab therapy in Chinese HCC patients.

## 2 Materials and methods

### 2.1 Collection of patient samples

In the present study, a total of 254 primary liver cancer patients were enrolled, including 232 HCC and 22 ICC samples, respectively. The clinical stages of patients ranged from Ⅰ to Ⅳ, which were defined according to the American Joint Committee on Cancer (Eighth Edition). Patient samples, together with clinicopathological information (such as diagnosis age, gender, clinical stage, serum α-fetoprotein (AFP), des-γ-carboxy-pro-thrombin (DCP), viral status, and survival time), were collected between 27th April 2015 and 18th April 2023, and written informed consents of all involved patients were obtained. This study was approved by the Ethics Committee of the Third Affiliated Hospital of Second Military Medical University (NCT03794440). Moreover, it was claimed that this study was conducted according to the Declaration of Helsinki and the International Ethical Guidelines for Biomedical Research Involving Human Subjects. The formalin-fixed, paraffin-embedded (FFPE) tumor samples were collected and subsequently evaluated by a qualified pathologist to confirm that tumor content (at least containing 20% tumor cells) was sufficient. Qualified samples were finally sent for targeted sequencing (a 603-cancer-gene panel, this gene list included the hepatobiliary cancer-related genes (*VEGFA*, *KRAS*, *NRAS*, *HRAS*, *MET*, *TP53*, *WNK2*, *CTNNB1*, and *TSC1*), DNA damage repair-associated genes (*BRCA1*, *BRCA2*, *BARD1*, and *BLM*), tumor immune-related genes (*CD274*, *CDKN2A*, *CDKN2B*, *CHEK1*, *CHEK2*, *CTNNB1*, and *DNMT3A*), genetic alteration-related genes (*APC*, *ARAF*, *ARID1A*, *JAK1*, *JAK2*, *JAK3*, *JUN*, *KDM4C*, *KDM5A*, *KDM5C*, *KDM5D*, *KDM6A*, *NOTCH1*, *NOTCH2*, *NOTCH3*, and *NOTCH4*), chemotherapy response-related genes (*ABCB1*, *ABCC2*, *CASP7*, *CBR3*, and *CDA*), and genetic predisposition-related genes (*APC*, *ATM*, *ATR*, *AXIN2*, *BAP1*, *BLM*, *BRCA1*, and *BRCA2*).) using the next-generation sequencing technology.

### 2.2 Library preparation and targeted sequencing

The FFPE tumor samples were extracted according to the instructions of the manufacturer using the QIAamp Genomic DNA Kit (QIAGEN, Hilden, Germany). The quality and quantity of purified tumor genomic DNA (gDNA) were analyzed using an Agilent 2100 Bioanalyzer (Agilent Technologies, CA, United States) and a Qubit 3.0 Fluorometer (Thermo Fisher Scientific Life Technologies, MA, United States). Next, 50 ng of gDNA was sheared using the Covaris E210 System (Covaris, MA, United States), and library construction was prepared using the Accel-NGS^®^ 2S Plus DNA Library Kit (Swift Biosciences, MI, United States). The gene probes (a 603-cancer-gene panel) were synthesized by Integrated DNA Technologies Inc. (MI, United States), and the targeted gene library was constructed using the ThruPLEX^®^ DNA-Seq Kit (TaKaRa Biomedical Technology Co., Ltd, Beijing, China). The constructed library was sequenced using the Illumina NovaSeq 6000 platform (Illumina, CA, United States), and paired-end sequencing was conducted with 150bp of length per read. The sequencing coverage must be 1,000× at least.

### 2.3 Variant calling and interpretation

After sequencing, FASTQ files were generated using software fastp, and quality control was conducted using Cassava. The raw data of sequencing reads were aligned to the Human Genome Reference (hg19) using the Burrows–Wheeler alignment tool, and binary sequence alignment map (BAM) files were finally acquired. Picard (http://broadinstitute.github.io/picard/) was subsequently used for duplicate removal and local realignment. Local realignment and base quality score recalibration were conducted using software GATK (http://software.broadinstitute.org/gatk). Variant calling, including single-nucleotide variants (SNVs) and small insertions or deletions (indels), was identified using MuTect2 and then annotated using software ANNOVAR. The alterations were interpreted by a molecular geneticist according to the guidelines and standards of the Association for Molecular Pathology, the American Society of Clinical Oncology, and the American College of Medical Genetics and Genomics. The calculation of TMB was conducted by counting the number of identified variants per megabase.

### 2.4 Clinicopathological and genomic data of Western HCC patients

The clinicopathological features and genomic data of HCC from TCGA were downloaded via cBioPortal (https://www.cbioportal.org/) to compare the genomic differences between Chinese and Western HCC patients. Of note, the retrieved TCGA HCC cohort totally comprised 360 HCC patients (samples without complete information were excluded); after Asian cases were filtered, the remaining 185 patients were regarded as Western HCC patients. In addition, a 603-cancer-gene panel was further utilized to filter genomic alterations in Western HCC patients.

### 2.5 Genomic alteration enrichment and survival analysis

Genomic profiling was visualized and performed to exhibit the differences in genomic alterations between different groups via the OncoPrint figures using the package maftools. Genomic alteration enrichment analysis (genomic alterations with alteration frequency <5.00% were not included in the subsequent statistical analysis) was conducted to identify the prevalently altered genes in each group. When grouping alterations by the signaling pathway, pathway-related alterations were determined according to the previously reported study totally defining 10 oncogenic signaling pathways (Sanchez-Vega et al., 2018), including Wnt, Hippo, Notch, RTK/RAS, PI3K, MYC, TGF-β, TP53, cell cycle, and NRF2 signaling pathways. In addition, univariate Cox regression analysis was conducted to identify OS or RFS-associated genomic alterations using the package rms. Multivariate Cox regression analysis was employed to determine the independent prognostic factors, including genomic alterations and clinicopathological features, using the package rms. Survival analysis using the Kaplan–Meier curve was conducted using the packages survival and survminer.

### 2.6 Sintilimab plus bevacizumab in unresectable/metastatic HCC

Of the 232 HCC patients enrolled in this study, 47 unresectable/metastatic HCC patients were further recommended for first-line treatment. These HCC patients were treated with sintilimab (an anti-PD-1 antibody, Xinda Biopharmaceutical (Suzhou) Co., Ltd, Jiangsu, China) and bevacizumab (an anti-VEGF antibody, Xinda Biopharmaceutical (Suzhou) Co., Ltd, Jiangsu, China). Eligible patients for this study were defined according to the following criteria (Finn et al., 2020): (ⅰ) patient age >18 years old; (ⅱ) Eastern Cooperative Oncology Group (ECOG) performance status: from 0 to 1; (ⅲ) patients had not previously received systemic therapy; (ⅳ) patients had measurable diseases as defined by Response Evaluation Criteria in Solid Tumors, version 1.1 (RECIST 1.1); (ⅴ) Child–Pugh classification A liver function; and (ⅵ) adequate hematologic and organ function. The key exclusion criteria mainly included (ⅰ) patients with a history of autoimmune disease; (ⅱ) fibrolamellar or sarcomatoid HCC or mixed HCC and cholangiocarcinoma type; (ⅲ) patients co-infected with HBV and HCV; and (ⅳ) patients with gastric varices at risk of bleeding or with high risk for bleeding and/or untreated or incompletely treated esophageal cancer. Magnetic resonance imaging (MRI) and/or computed tomography (CT) scans were reviewed and evaluated by a qualified radiologist. The primary endpoints were OS and progression-free survival (PFS). The criteria of therapy response were defined as follows: CR, complete response; PR, partial response; SD, stable disease; and PD, progressive disease. The objective response rate was the proportion of patients with CR/PR. Durable clinical benefit (DCB) was the percentage of patients with CR/PR/SD, while non-DCB (NDB) was the percentage of patients with PD.

### 2.7 Statistical analysis

The statistical analysis was conducted using the chi-squared test, Fisher’s exact test, Mann–Whitney U test, and Kruskal–Wallis test in Rstudio (https://rstudio.com/). Survival analysis was conducted using the Kaplan–Meier curve, and the log-rank test was used for comparison. Hazard ratios were calculated by Cox regression analysis. A two-sided *p*-value less than 0.05 (*p* < 0.05) was considered statistically significant.

## 3 Results

### 3.1 Clinicopathological features of Chinese HCC/ICC patients

**FIGURE 1:**
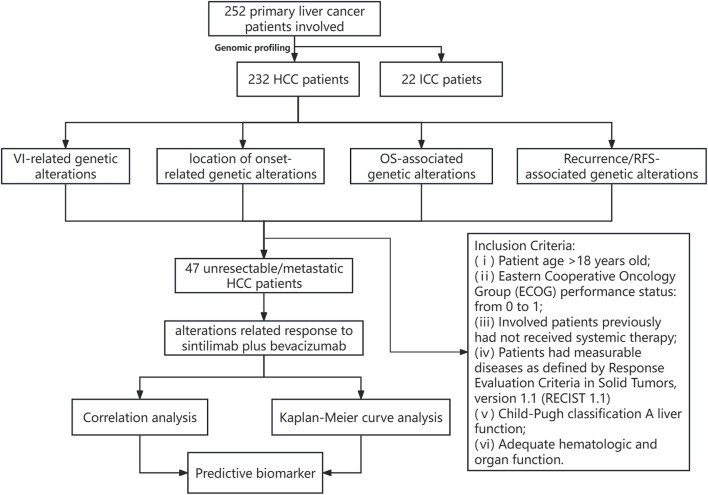
Flow chart of this study.

Detailed clinicopathological information is provided in Table 1, including 232 HCC patients and 22 ICC patients. Among HCC patients, there were 209 males and 23 females, and the median diagnosis age was 56 years (ranging from 30 to 80 years old); among ICC patients, there were 13 males and 9 females, and the median diagnosis age was 59 years (ranging from 37 to 74 years old). Among HCC patients, 220 were infected with HBV and 4 were infected with HBV&HCV; while 17 ICC patients were ever infected with HBV, no one was infected with HCV or HBV&HCV. Moreover, 119 and 18 patients had HCC and ICC recurrences, respectively. The median TMB of HCC vs. ICC was 8.7 [0.6, 25] vs. 5.3 [0.7, 11]. Further statistical analysis revealed that a significantly higher proportion of HCC patients were male (90.09% vs. 59.09%, *p* < 0.01) and more HCC patients were infected with HBV (94.82% vs. 77.27%, *p* < 0.05) compared to ICC patients. Nevertheless, ICC patients had a higher proportion of patients with lymph node metastasis (59.09% vs. 2.16%) and a markedly higher recurrence rate (81.82% vs. 51.29%, *p* < 0.05) than HCC patients in our cohort. The flow chart of this study is shown in Figure 1.

**TABLE 1:** Clinicopathological features in the Chinese HCC/ICC cohort.

Variable		HCC	ICC	*p*-value
Total		232	22	
Diagnosis age	Median (range)	56 [30, 80]	59 [37, 74]	= 0.33
Gender	Male	209	13	<0.01
	Female	23	9	
Clinical stage	Ⅰ	94	10	= 0.44
	Ⅱ	54	2	
	Ⅲ	72	8	
	Ⅳ	12	2	
Vascular invasion	Yes	93	11	= 0.50
	No	139	11	
LNM	Yes	5	13	<0.01
	No	227	9	
RM	Yes	12	2	= 0.78
	No	220	19	
HBC/HCV infection	HBV-infected	220	17	<0.05
	HCV-infected	0	0	
	HBV- and HCV-infected	4	0	
	Not infected	8	5	
Location of onset	Left hepatic lobe	38	NA	NA
	Right hepatic lobe	146	NA	
	Other	48	NA	
Recurrence	Yes	119	18	<0.05
	No	113	4	
TMB	Median (range)	8.7 [0.6, 25]	5.3 [0.7, 11]	= 0.08

HCC, hepatocellular carcinoma; ICC, intrahepatic cholangiocarcinoma; LNM, lymph node metastasis; RM, remote metastasis; HBV/HCV, hepatitis B/C virus; TMB, tumor mutational burden; *p* < 0.05 was considered statistically significant.

### 3.2 Genomic landscape of Chinese HCC and ICC patients

**FIGURE 2:**
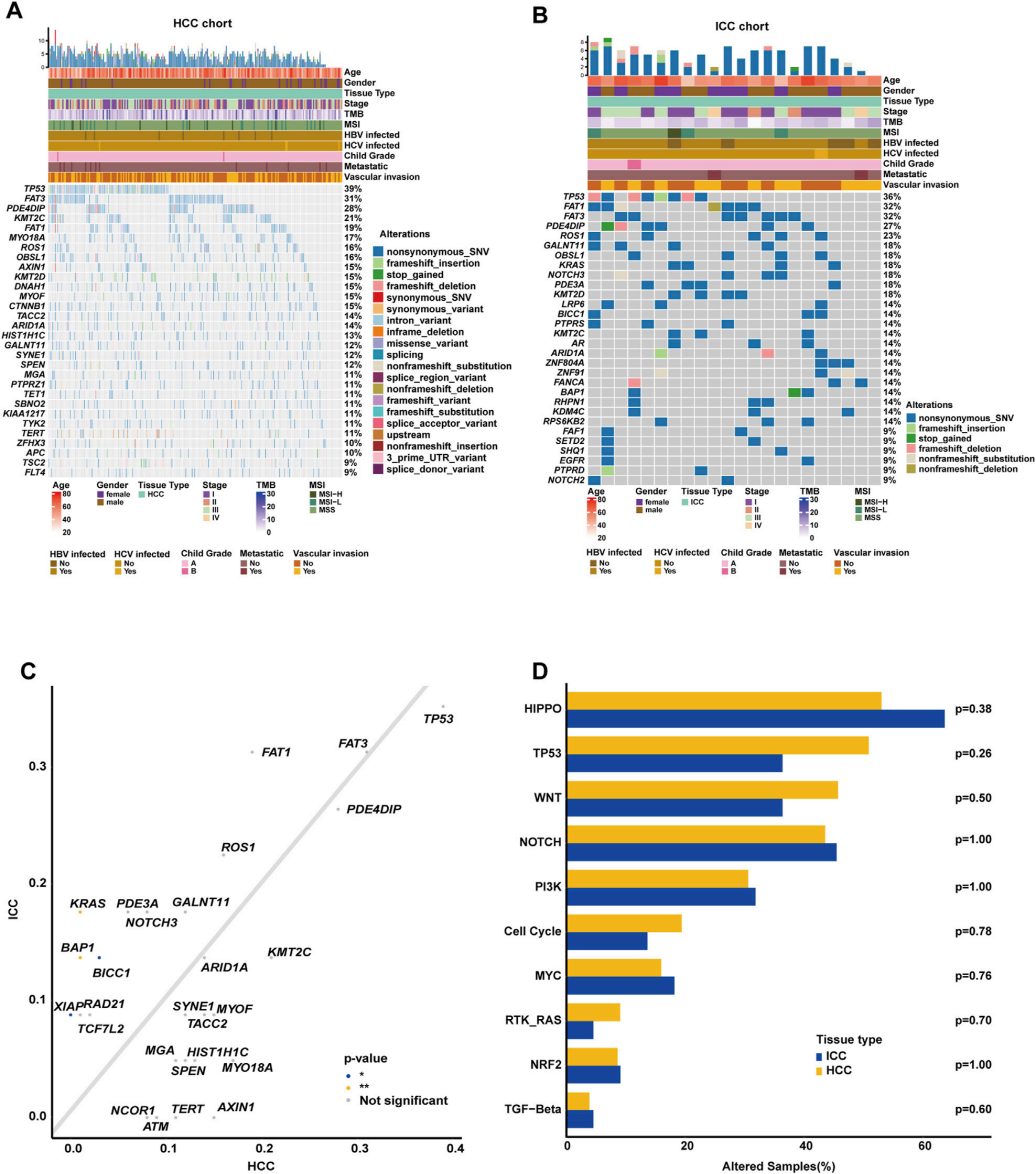
Comparison of genomic alterations between Chinese HCC and ICC patients. **(A)** OncoPrint plot exhibiting the genomic landscape of Chinese HCC. **(B)** OncoPrint plot demonstrating the genomic landscape of Chinese ICC. **(C)** Genomic alteration enrichment analysis between Chinese HCC and ICC. **(D)** Genomic enrichment analysis underlying 10 oncogenic signaling pathways between Chinese HCC and ICC.

In this Chinese HCC/ICC cohort, the genomic landscape of HCC patients (Figure 2A) exhibited the top 10 altered genes, namely, *TP53* (38.79%), *FAT3* (31.03%), *PDE4DIP* (27.59%), *KMT2C* (21.12%), *FAT1* (19.40%), *MYO18A* (16.81%), *ROS1* (16.38%), *OBSL1* (15.95%), *AXIN1* (15.09%), and *KMT2D* (15.09%). Of note, the alteration frequencies of *CTNNB1* and *TERT* were only 14.66% and 10.78%, respectively, in our HCC cohort. The genomic landscape of ICC patients (Figure 2B) displayed that *TP53* (36.36%), *FAT1* (31.82%), *FAT3* (31.82%), *PDE4DIP* (27.27%), *ROS1* (22.73%), *GALNT11* (18.18%), *OBSL1* (18.18%), *KRAS* (18.18%), *NOTCH3* (18.18%), and *PDE3A* (18.18%) were the most frequently altered genes. Impressively, aberrant regulation of *FAT3*, *KMT2C*, and *FAT1* was significantly correlated with the proliferation and migration of HepG2 and SMMC-7721 cell lines (Supplementary Figure S1). The comparison of genomic landscapes between HCC and ICC demonstrated that *KRAS* (18.18% vs. 1.29%, *p* < 0.01) and *BAP1* (13.64% vs. 1.29%, *p* < 0.01) alterations were more prevalent among Chinese ICC patients (Figure 2C). When grouping genomic alterations by signaling pathways (Figure 2D), it was observed that Hippo (53.02%), TP53 (50.86%), Wnt (45.69%), Notch (43.53%), PI3K (30.60%), and cell cycle (19.40%) were the top prevalently altered pathways in HCC; on the other hand, the top prevalently altered pathways in Chinese ICC included Hippo (63.64%), Notch (45.45%), TP53 (36.36%), Wnt (36.36%), PI3K (31.82%), and MYC (18.18%) signaling pathways. Further comparison revealed no statistically significant difference in the alteration frequency of signaling pathways between HCC and ICC.

### 3.3 Clinicopathological and genomic discrepancies between Chinese and Western HCC cohorts

**FIGURE 3:**
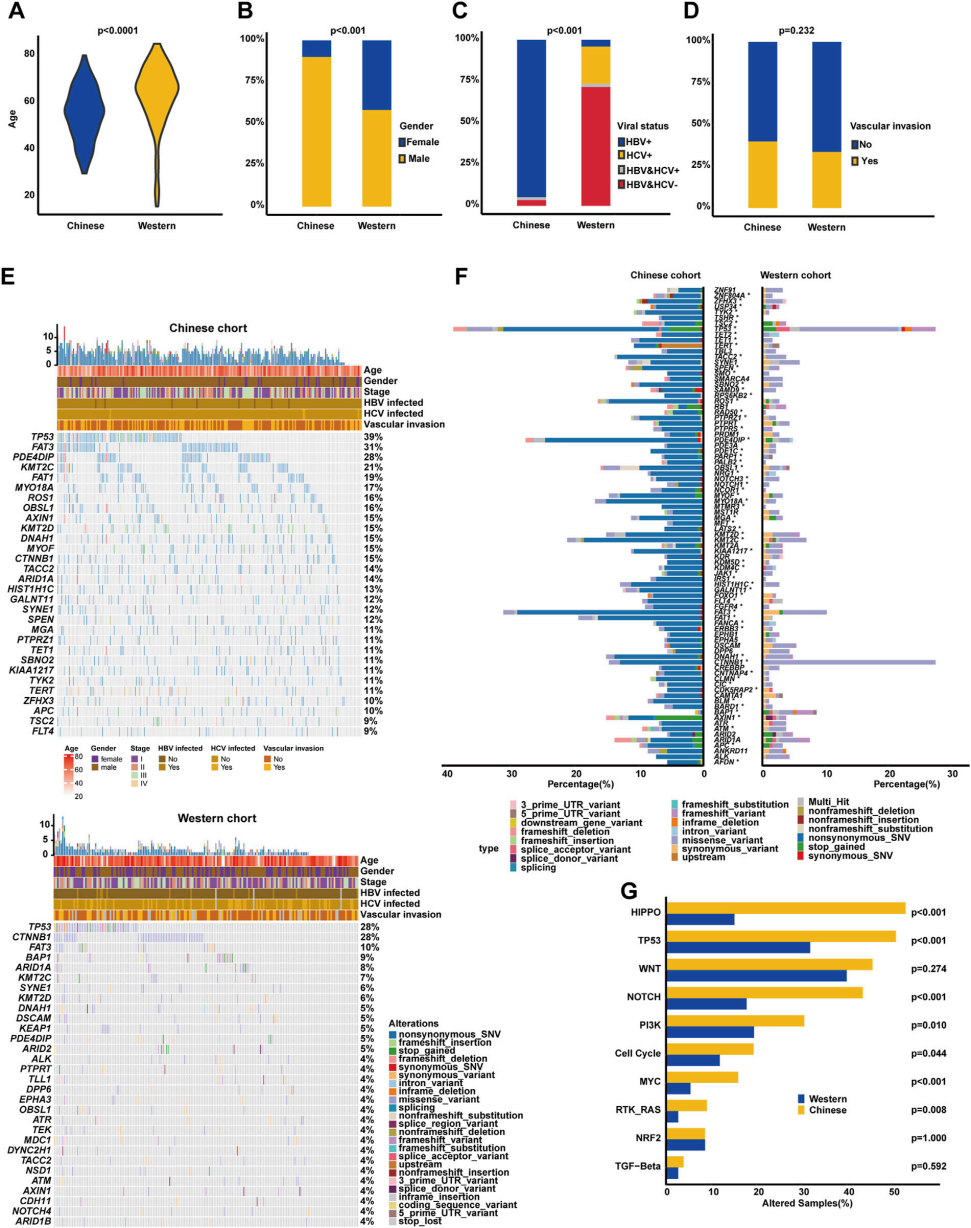
Clinicopathological and genomic comparison between Chinese and Western HCC cohorts. The difference analysis of diagnosis age **(A)**, the proportion of male and female cases **(B)**, viral status **(C)**, and the percentage of vascular invasion **(D)** between Chinese and Western HCC. **(E)** Genomic profiling showing the top altered genes based on a 603-cancer-gene panel, respectively, in Chinese and Western HCC cohorts. **(F)** The prevalently altered genes and alteration type were displayed, respectively, in Chinese and Western HCC cohorts. **(G)** Genomic enrichment analysis underlying 10 oncogenic signaling pathways between Chinese and Western HCC patients.

After excluding the Asian HCC patients, a total of 185 Western HCC patients from TCGA HCC cohort were eventually collected (Table 2). The comparison of clinicopathological variables between Chinese and Western HCC cohorts revealed that the patient diagnosis age (median age at diagnosis: 65 [16, 85]) in the Western HCC cohort was older than that (median age at diagnosis: 56 [30, 80]) in the Chinese HCC cohort (*p* < 0.0001, Figure 3A). Moreover, it was found that the Chinese HCC cohort had a relatively higher proportion of male patients (90.09% vs. 58.92%, *p* < 0.001, Figure 3B). Strikingly, over 90% of Chinese HCC patients were HBV carriers, but just 4.32%, 22.16%, and 2.16% of Western HCC patients had HBV, HCV, and HBV&HCV infection, respectively, (*p* < 0.001, Figure 3C). Regarding other clinicopathological phenotypes (Table 2), such as VI (Figure 3D), there was no statistically significant difference between Chinese and Western HCC cohorts.

**TABLE 2:** Clinicopathological comparison between Chinese and Western HCC patients.

Variable		Chinese	Western	*p*-value
Total		232	185	
Diagnosis age	Median (range)	56 [30, 80]	65 [16, 85]	<0.01
Gender	Male	209	109	<0.01
	Female	23	76	
Clinical stage	Ⅰ	94	83	= 0.08
	Ⅱ	54	48	
	Ⅲ	72	42	
	Ⅳ	12	3	
	Unknown	0	9	
Vascular invasion	Yes	93	57	= 0.23
	No	139	112	
	Unknown	0	16	
LNM	Yes	5	3	= 0.18
	No	227	96	
	Unknown	0	86	
RM	Yes	12	2	= 0.26
	No	220	105	
	Unknown	0	78	
HBV/HCV infection	HBV-infected	220	8	<0.01
	HCV-infected	0	41	
	HBV- and HCV-infected	4	4	
	Not infected	8	132	

HCC, hepatocellular carcinoma; LNM, lymph node metastasis; RM, remote metastasis; HBV/HCV, hepatitis B/C virus; *p* < 0.05 was considered statistically significant.

Subsequently, the comparison of genomic landscapes directly exhibited that there existed great discrepancies in genomic profiling (Figure 3E) between Chinese and Western HCC cohorts. Statistical analysis (Figure 3F) identified that alteration frequencies of *TP53* (38.79% vs. 27.57%), *FAT3* (31.03% vs. 10.27%), *PDE4DIP* (27.59% vs. 4.86%), *KMT2C* (21.12% vs. 7.03%), *FAT1* (19.40% vs. 1.62%), *MYO18A* (16.81% vs. 2.16%), *ROS1* (16.38% vs. 2.70%), *OBSL1* (15.95% vs. 4.32%), *AXIN1* (15.09% vs. 3.78%), and *KMT2D* (15.09% vs. 5.95%) were all significantly higher in the Chinese HCC cohort than those in the Western HCC cohort. On the other hand, it was noticed that *CTNNB1* (27.57% vs. 14.66%) and *BAP1* (8.65% vs. 1.29%) were prevalently altered in the Western HCC cohort. Noticeably, the genomic alteration type of Chinese HCC patients was mainly enriched in nonsynonymous SNV; however, the Western HCC cohort had more alterations enriched in the genomic alteration type of the missense variant (Figure 3F). When grouping genomic alterations by signaling pathways, Hippo (53.02% vs. 15.14%), TP53 (50.86% vs. 31.89%), Notch (43.53% vs. 17.84%), PI3K (30.60% vs. 19.46%), cell cycle (19.40% vs. 11.89%), MYC (15.95% vs. 5.41%), and RTK/RAS (9.05% vs. 2.70%) pathway-related alterations were more prevalent in the Chinese HCC cohort (Figure 3G).

### 3.4 Genomic profiling underlying vascular invasion

**FIGURE 4:**
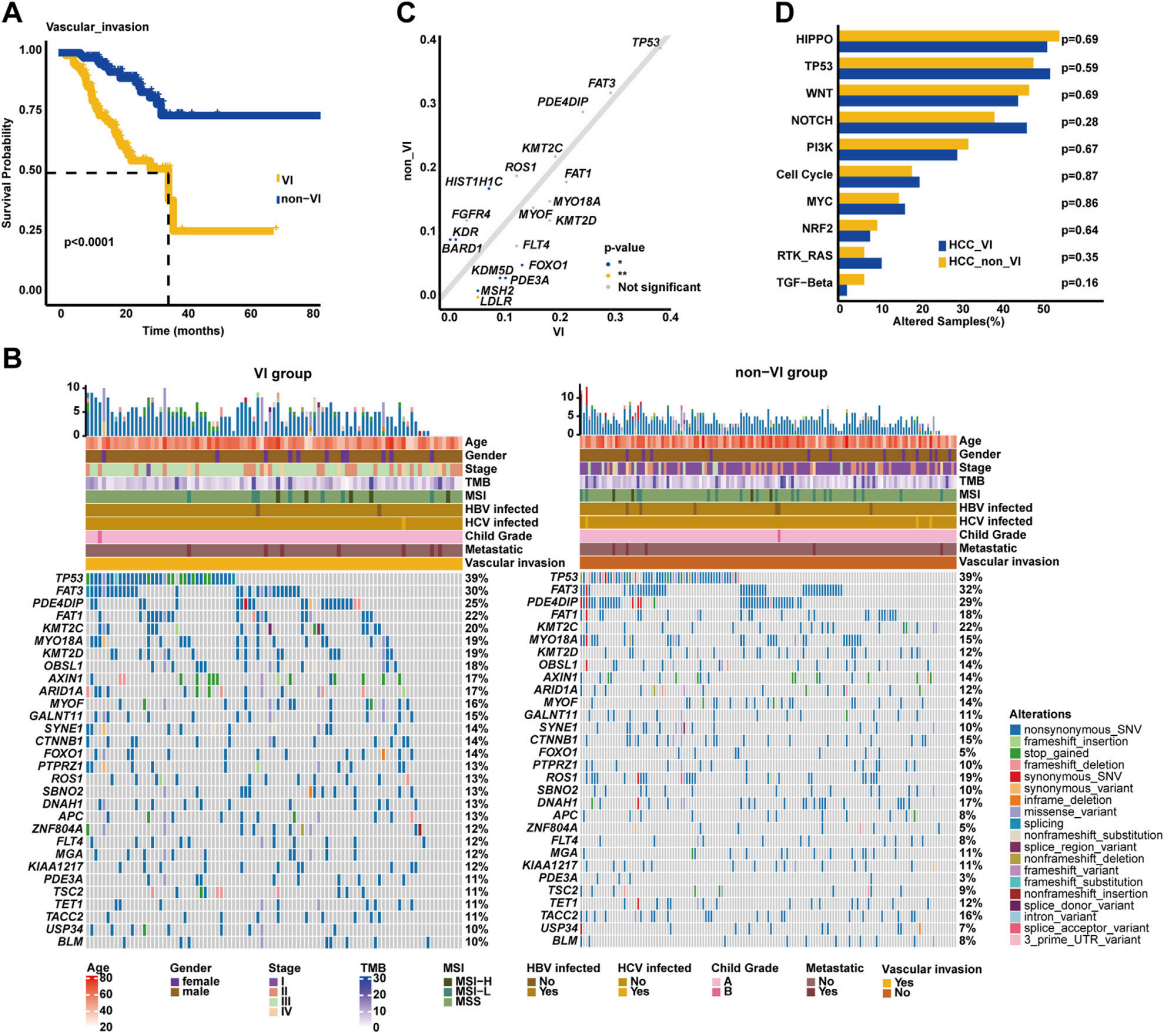
Genomic profiling underlying VI in the Chinese HCC cohort. **(A)**. Kaplan–Meier curve analysis of OS between VI and non-VI groups. **(B)** The most commonly altered genes were exhibited, respectively, in the VI and non-VI groups. **(C)** Genomic enrichment analysis identified the frequently altered genes in the VI group. **(D)** Prevalent signaling pathway-related alterations, respectively, in the VI and non-VI groups.

It is known that VI is usually associated with the progression of HCC and negatively correlated with the prognosis of HCC. In our cohort, it was similarly found that HCC patients with VIs had inferior OS (median OS: 35.23 months vs. unreached, *p* < 0.0001, Figure 4A) compared to those without VIs. Of note, there were some genomic differences between the VI and non-VI groups (Figure 4B). The genomic landscape exhibited that *TP53* (38.71%), *FAT3* (30.11%), *PDE4DIP* (24.73%), *FAT1* (21.51%), *KMT2C* (20.43%), *MYO18A* (19.35%), *KMT2D* (19.35%), *OBSL1* (18.28%), *AXIN1* (17.20%), and *ARID1A* (17.20%) were the top 10 altered genes in the VI group; on the other hand, the top 10 prevalent genes in the non-VI group included *TP53* (38.85%), *FAT3* (31.65%), *PDE4DIP* (29.50%), *KMT2C* (21.58%), *ROS1* (18.71%), *FAT1* (17.99%), *HIST1H1C* (16.55%), *DNAH1* (16.55%), *TACC2* (15.83%), and *CTNNB1* (15.11%). Noticeably, it was further identified that *LDLR* (6.45% vs. 0.00%), *MSH2* (6.45% vs. 0.72%), *KDM5D* (9.68% vs. 2.88%), *PDE3A* (10.75% vs. 2.88%), and *FOXO1* (13.98% vs. 5.04%) alteration frequencies were higher in the VI group (*p* < 0.05, Figure 4C), while *HIST1H1C* (16.55% vs. 7.53%), *KDR* (8.63% vs. 2.15%), and *BARD1* (8.63% vs. 2.15%) were frequently altered in the non-VI group. However, there was no statistically significant difference observed in the alteration frequency of signaling pathways between VI and non-VI groups (Figure 4D).

### 3.5 Genomic differences between the left and right hepatic lobes of HCC

**FIGURE 5:**
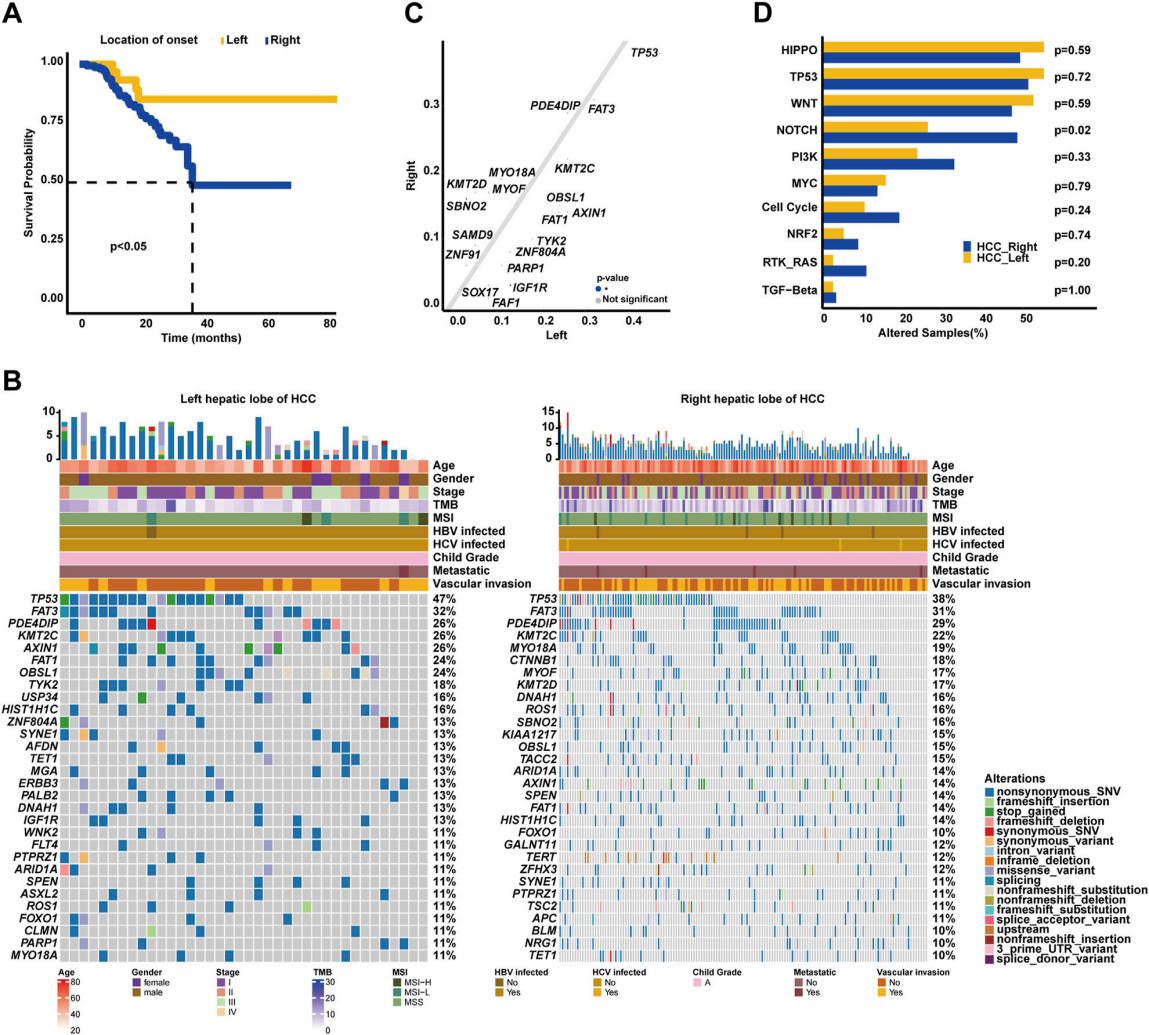
Association of genomic characterizations with the location of onset in HCC. **(A)** Kaplan–Meier curve analysis of OS between the left and right hepatic lobes of HCC. **(B)** Genomic landscape of left and right hepatic lobes of HCC. **(C)** Genomic alteration enrichment analysis between the left and right hepatic lobes of HCC. **(D)** Comparison analysis of alterations in oncogenic signaling pathways between the left and right hepatic lobes of HCC.

Intriguingly, it was identified in our cohort that HCC patients with tumors located in the left hepatic lobes had superior OS (median OS: 36.77 months vs. unreached, *p* < 0.05, Figure 5A) than those with tumors in the right hepatic lobes. The most prevalently altered genes among the left hepatic lobes of HCC patients included *TP53* (47.37%), *FAT3* (31.58%), *PDE4DIP* (26.32%), *KMT2C* (26.32%), *AXIN1* (26.32%), *FAT1* (23.68%), *OBSL1* (23.68%), *TYK2* (18.42%), *USP34* (15.79%), and *HIST1H1C* (15.79%), whereas the right hepatic lobes of the HCC group had the enrichment of alterations in *TP53* (38.36%), *FAT3* (30.82%), *PDE4DIP* (29.45%), *KMT2C* (21.92%), *MYO18A* (19.18%), *CTNNB1* (17.81%), *MYOF* (17.12%), *KMT2D* (17.12%), *DNAH1* (16.44%), and *ROS1* (15.75%) genes (Figure 5B). Subsequent analysis showed that *SBNO2* (15.75% vs. 2.63%) was prevalently altered in the right hepatic lobes of the HCC group; in comparison, *IGF1R* alterations (13.16% vs. 3.42%) were more frequent in the left hepatic lobes of the HCC group (*p* < 0.05, Figure 5C). When grouping genomic alterations by functional significance, notably, Notch signaling pathway-related alterations (48.63% vs. 26.32%, *p* < 0.05, Figure 5D) were prevalent among the right hepatic lobes of HCC patients.

### 3.6 Effects of genomic alterations on overall survival

**FIGURE 6:**
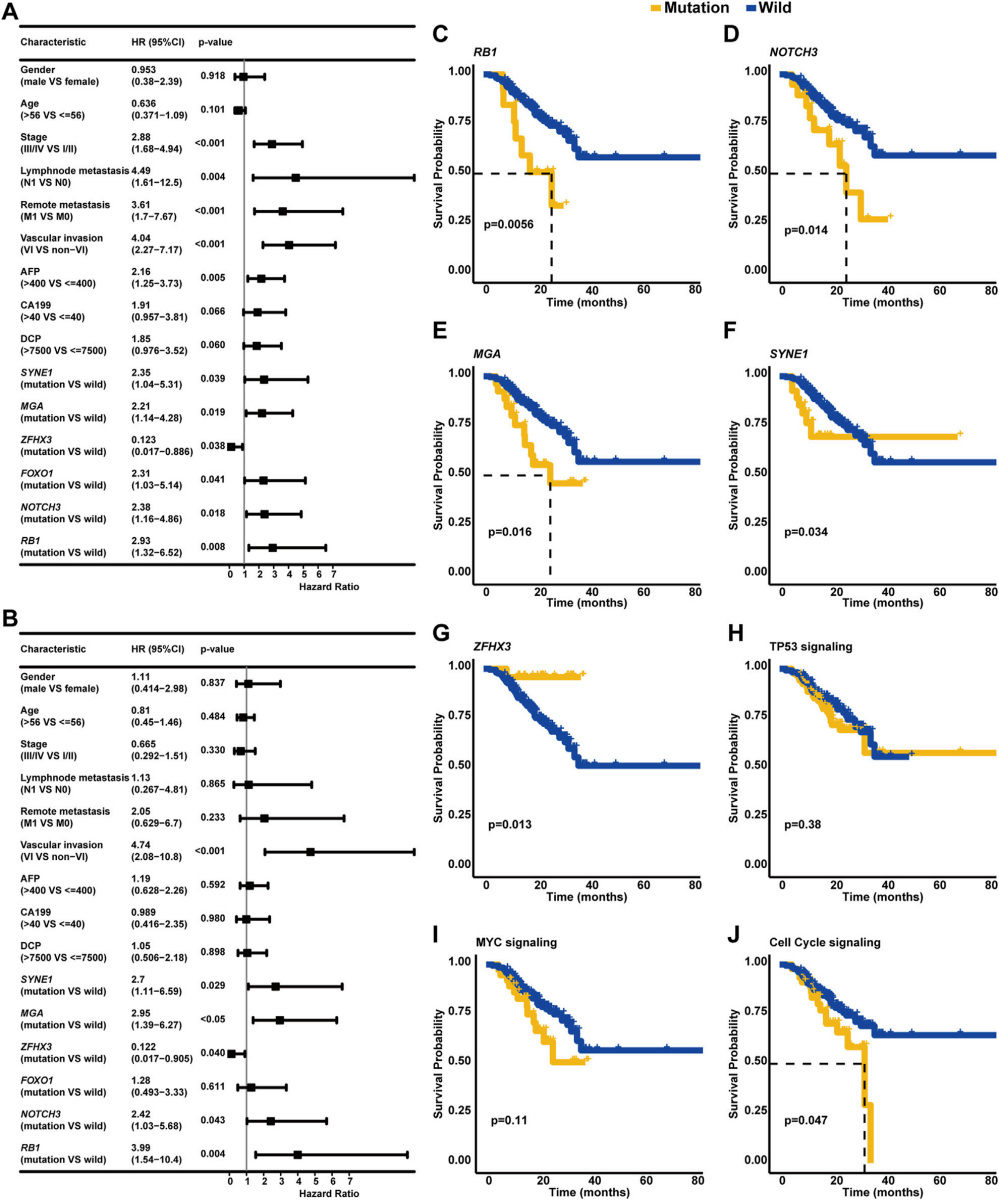
Identification of clinicopathological and genetic factors influencing the OS of HCC. **(A)** The univariate Cox regression analysis discovered the risk or protective factors in HCC. **(B)** Multivariate Cox regression analysis was performed to identify the independent biomarkers in predicting the OS of HCC. The Kaplan–Meier curve analysis was further conducted to compare the OS of wild-type patients and individuals with *RB1* **(C)**, *NOTCH3* **(D)**, *MGA* **(E)**, *SYNE1* **(F)**, *ZFHX3* **(G)**, TP53 signaling, **(H)** MYC signaling **(I)**, cell cycle signaling, or **(J)** related mutations.

In addition to clinical stage, lymph node metastasis, remote metastasis, VI, and AFP level, the univariate Cox regression analysis further identified that altered genes *SYNE1*, *MGA*, *FOXO1*, *NOTCH3*, and *RB1* were also risk factors (*p* < 0.05, Figure 6A) in our cohort; on the contrary, *ZFHX3* alterations were positively correlated with OS. Strikingly, the multivariate Cox regression analysis revealed that VI and altered genes *SYNE1*, *MGA*, *NOTCH3*, and *RB1* were independent risk factors, while the altered gene *ZFHX3* was an independent protective factor for HCC patients (*p* < 0.05, Figure 6B). The Kaplan–Meier curve analysis exhibited that HCC patients with altered *RB1*, *NOTCH3*, *MGA*, or *SYNE1* genes had inferior OS (median OS of altered type vs. wild type: *RB1*: 26.37 months vs. unreached, *p* < 0.01, Figure 6C; *NOTCH3*: 25.83 months vs. unreached, *p* < 0.05; Figure 6D; and *MGA*: 25.83 months vs. unreached, *p* < 0.05; Figure 6E; and *SYNE1*: unreached vs. unreached, *p* < 0.05; Figure 6F) compared to patients with wild-type genes *RB1*, *NOTCH3*, *MGA*, or *SYNE1*, respectively. In addition, the Kaplan–Meier curve analysis displayed that HCC patients with *ZFHX3* alterations had superior OS (median OS: unreached vs. unreached, *p* < 0.05, Figure 6G). Regarding the effects of signaling pathway-related genomic alterations on the OS of HCC patients, it was identified that HCC patients with alterations in TP53 or MYC signaling pathways had slightly worse OS (median OS of altered type vs. wild type: TP53 signaling: unreached vs. unreached, *p* = 0.38, Figure 6H; MYC signaling: unreached vs. unreached, *p* = 0.11; Figure 6I) than those without associated alterations. Of note, HCC patients with cell cycle-associated alterations had significantly worse OS (median OS: 32.9 months vs. unreached, *p* < 0.05, Figure 6J) than individuals with wild-type alterations.

### 3.7 Association of clinicopathological/genomic features with recurrence

**FIGURE 7:**
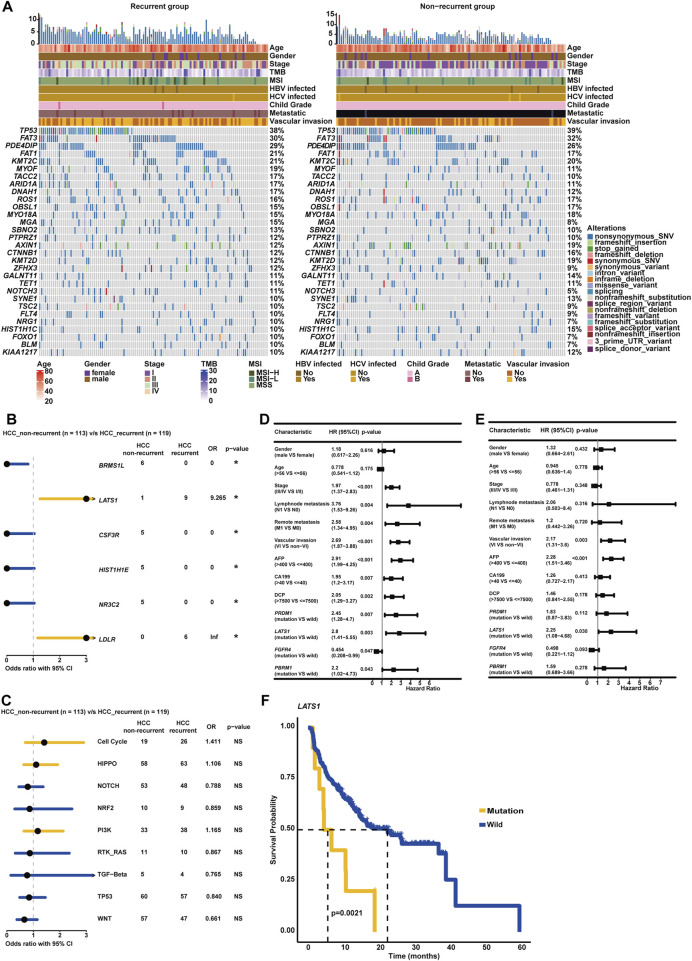
Genomic profiling deciphered specific molecular characterizations correlated with recurrence and RFS in HCC. **(A)** Genomic landscape of HCC-recurrent and non-recurrent groups. **(B)** The forest plot exhibited enrichment of genomic alterations, respectively, in HCC-recurrent and non-recurrent groups. **(C)** Genomic enrichment analysis underlying oncogenic signaling pathways between HCC-recurrent and non-recurrent groups. **(D)** The univariate Cox regression analysis was conducted to distinguish the risk or protective factors influencing the RFS of HCC. **(E)** The multivariate Cox regression analysis was performed to identify the independent biomarkers in predicting the RFS of HCC. **(F)** Altered *LATS1* was markedly associated with the RFS of HCC.

In this study, recurrence was observed in over 50% of HCC patients (119/232), and clinicopathological association analysis uncovered that HCC recurrence was significantly associated with advanced clinical stage, VI, lymph node metastasis, and remote metastasis (*p* < 0.05, Table 3). Among these recurrent HCC patients, the genomic landscape manifested that *TP53* (38.24%), *FAT3* (29.83%), *PDE4DIP* (28.99%), *FAT1* (21.42%), *KMT2C* (20.59%), *MYOF* (18.91%), *TACC2* (17.22%), *ARID1A* (17.22%), *DNAH1* (17.22%), and *ROS1* (16.39%) genes alterations were the most commonly altered (Figure 7A). Of note, it was found that *LATS1* (7.56% vs. 0.88%) and *LDLR* (5.06% vs. 0.00%) alterations were prevalent in the recurrent HCC group (*p* < 0.05, Figure 7B). When grouping genomic alterations by signaling pathways, HCC recurrence seemed not to be associated with signaling pathway-related alterations (Figure 7C). Additionally, the univariate Cox regression analysis identified that clinical stage, lymph node metastasis, remote metastasis, VI, AFP level, CA199 level, and DCP level, and altered genes *PRDM1*, *LATS1*, *FGFR4*, and *PBRM1* were significantly correlated with the RFS of HCC patients (*p* < 0.05, Figure 7D). Strikingly, multivariate Cox regression analysis revealed that in addition to clinicopathological features of VI and AFP level, only the *LATS1* gene as the risk factor was independent in predicting RFS for HCC patients (*p* < 0.05, Figure 7E). The Kaplan–Meier curve analysis further showed that HCC patients with altered *LATS1* had the worse RFS (median RFS: 5.57 months vs. 22.47 months, *p* < 0.01, Figure 7F).

**TABLE 3:** Clinicopathological comparison of HCC according to recurrence.

Variable		Recurrence	No recurrence	*p*-value
Total		119	113	
Diagnosis age	Median (range)	55 [30, 79]	57 [31, 80]	= 0.32
Gender	Male	109	100	= 0.43
	Female	10	13	
Clinical stage	Ⅰ	33	61	<0.01
	Ⅱ	32	22	
	Ⅲ	44	28	
	Ⅳ	10	2	
Vascular invasion	Yes	64	29	<0.01
	No	55	84	
LNM	Yes	5	0	<0.05
	No	114	113	
RM	Yes	10	2	<0.05
	No	109	111	
HBC/HCV infection	HBV-infected	115	105	= 0.41
	HCV-infected	0	0	
	HBV- and HCV-infected	1	3	
	Not infected	3	5	
Location of onset	Left hepatic lobe	16	22	= 0.23
	Right hepatic lobe	74	72	
	Other	29	19	
TMB	Median (range)	6.1 [0.6, 25]	6.5 [2.1, 23]	= 0.18

HCC, hepatocellular carcinoma; LNM, lymph node metastasis; RM, remote metastasis; HBV/HCV, hepatitis B/C virus; TMB, tumor mutational burden; *p* < 0.05 was considered statistically significant.

### 3.8 Evaluation of sintilimab plus bevacizumab treatment response

**FIGURE 8:**
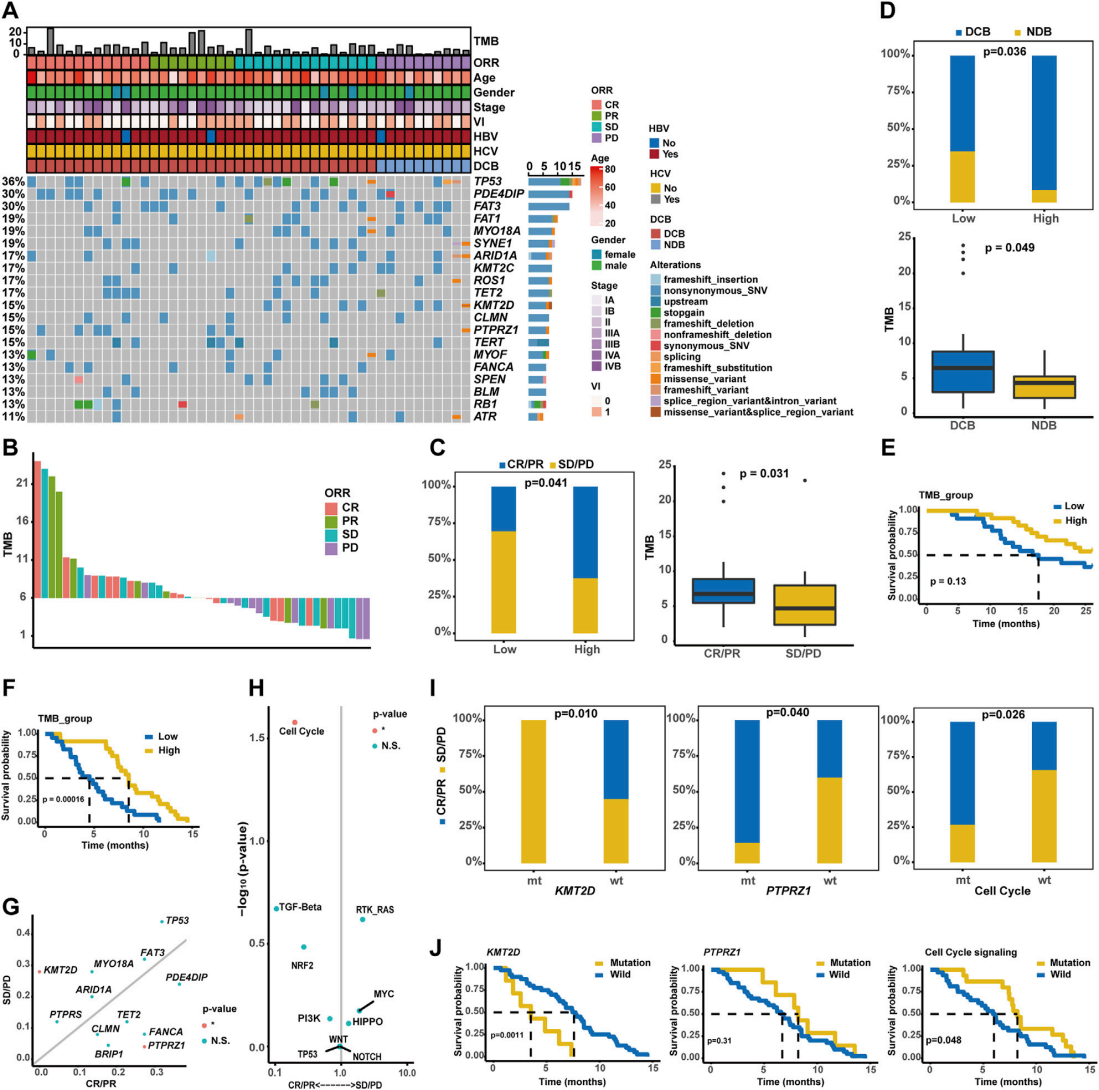
Estimate of sintilimab (anti-PD-1) plus bevacizumab therapy response in unresectable/metastatic HCC patients. **(A)** Distribution of genomic alterations correlated with combination immunotherapy. **(B)** The waterfall plot exhibited the distribution of CR, PR, SD, and PD patients across varied TMB values. **(C)** Association of TMB values with ORR. **(D)** Association of TMB values with DCB. **(E)** Kaplan–Meier curve analysis of OS between TMB-high and TMB-low groups. **(F)** Comparison of time to progression between the TMB-high and TMB-low groups. **(G)** Genomic alteration enrichment identified *KMT2D* and *PTPRZ1* genes prevalently altered in SD/PD and CR/PR groups, respectively. **(H)** Alterations in the signaling pathways prevalent in SD/PD and CR/PR groups. **(I)** Association of *KMT2D*, *PTPRZ1*, or cell cycle signaling-related alterations with ORR. **(J)** Kaplan–Meier curve analysis of PFS between wild-type patients and cases with alterations in the *KMT2D*, *PTPRZ1*, or cell cycle signaling.

As described, a total of 47 unresectable/metastatic HCC patients involved in the present study received the combination therapy of sintilimab plus bevacizumab, and baseline characteristics are provided in Table 4. Initially, the OncoPrint plot exhibited the distribution of clinicopathological/genomic variations associated with the response of combination therapy (Figure 8A). It was noticed that all *KMT2D* alterations occurred in the CR/PR group, but a majority of *PTPRZ1* alterations were enriched in the SD/PD group. The waterfall plot showed that higher TMB values were associated with a larger proportion of patients with CR/PR (Figure 8B). Here, 47 HCC patients were divided into TMB-high (n = 24) and TMB-low (n = 23) groups, respectively, according to the median TMB as the cut-off value. In the TMB-high group, there was a significantly higher proportion of patients with CR/PR (62.50% vs. 30.43%, *p* < 0.05, Figure 8C), and expectedly, patients with CR/PR had a higher TMB value (median TMB value: 6.9 vs. 5.0) than those with SD/PD. In addition, the TMB-high group had significantly more patients with DCB (91.67% vs. 65.22%, *p* < 0.05, Figure 8D) than the TMB-low group; accordingly, patients with DCB had a higher level of TMB (median TMB value: 6.8 vs. 4.3, *p* < 0.05) than those with NDB. Impressively, the TMB-high group had superior OS (median OS: unreached vs. 17.43 months, *p* = 0.07, Figure 8E), and importantly, the TMB-high group had significantly better PFS (median PFS: 8.52 months vs. 3.79 months, *p* < 0.001, Figure 8F) than the TMB-low group. In addition, genomic alteration enrichment analysis confirmed that *KMT2D* and *PTPRZ1* alterations were significantly enriched in the SD/PD and CR/PR groups, respectively (Figure 8G), and it was further identified that cell cycle-related alterations were abundantly enriched in the CR/PR group (Figure 8H). A markedly higher proportion of patients with CR/PR (55.00% vs. 0.00%, *p* < 0.05, Figure 8I) were enriched in the wild-type *KMT2D* group, whereas altered *PTPRZ1* (85.71% vs. 40.00%, *p* < 0.05) or cell cycle signaling (73.33% vs. 36.67%, *p* < 0.05) group had the enrichment of patients with CR/PR.

**TABLE 4:** Baseline characteristics of unresectable/metastatic HCC.

Variable		Patient’s sample
Total		47
Diagnosis age	Median (range)	57 [30, 79]
Gender	Male	42
	Female	5
Clinical stage	Ⅰ	13
	Ⅱ	13
	Ⅲ	11
	Ⅳ	10
Vascular invasion	Yes	22
	No	25
LNM	Yes	3
	No	44
RM	Yes	10
	No	37
HBC/HCV infection	HBV-infected	44
	HCV-infected	0
	Not infected	3
TMB	Median (range)	6.1 [0.6, 24]

HCC, hepatocellular carcinoma; LNM, lymph node metastasis; RM, remote metastasis; HBV/HCV, hepatitis B/C virus; TMB, tumor mutational burden.

Notably, no significant difference in OS was observed between the altered (*KMT2D*, *PTPRZ1*, or cell cycle signaling pathways) and wild-type groups (Supplementary Figure S2), but patients with *KMT2D*, *PTPRZ1*, or cell cycle signaling-related alterations had markedly differential PFS (median PFS of altered type vs. wild-type: *KMT2D*: 2.56 months vs. 7.59 months, *p* < 0.01; *PTPRZ1*: 8.21 months vs. 6.70 months, *p* = 0.31; and cell cycle: 8.21 months vs. 5.98 months, *p* < 0.05, Figure 8J). The representative PD-1 immunohistochemistry staining is shown in Supplementary Figure S3, and it was further obtained that the characterizations of TMB-high, wild-type *KMT2D*, altered *PTPRZ1*, and altered cell cycle signaling were positively associated with the PD-1 expression (Supplementary Figures S4, S5).

## 4 Discussions

Liver cancer is still a global health problem with elevated incidence and mortality (Sung et al., 2021). Nowadays, multiple types of morphological/clinicopathological classifications, such as ⅰ) HCC, ICC, and mixed subtype; ⅱ) clinical staging; and ⅲ) tumor, lymph node, and metastasis (TNM) staging, have been applied as mainstream methods in clinical practice to help improve the diagnosis and therapeutic intervention for patients with liver cancer. Since the eighth edition of the cancer staging manual promulgated by the American Joint Committee on Cancers, precision molecular oncology and targeted therapy have been highlighted to substitute a more personalized approach for a population-based method (Amin et al., 2017). In the past few years, advances in the field of cancer genomics have revolutionized molecular characterization, which has been promoting the clinical management and development of precision medicine for a variety of cancer types (Chakravarty and Solit, 2021). In the present study, a larger prospective Chinese live cancer cohort study was conducted, and notably, genomic differences were further identified between Chinese HCC and ICC. The integrative analysis uncovered that clinicopathological and especially genomic characterizations were distinct between the Chinese and Western HCC cohorts. Genomic enrichment analysis exhibited that some specific genomic characterizations might be closely correlated with VI and the location of onset in HCC. Ultimately, this study deeply explored the association of genomic characterizations with recurrence, RFS, OS, and the response of anti-PD-1 plus bevacizumab in HCC. Overall, comprehensive genomic profiling will greatly help identify the potential distinct pathogenetic mechanisms, discover novel biomarkers, and offer matched or targeted therapeutic strategies effective for patients.

Initially, in the present study, the genomic landscape of HCC patients in our cohort displayed that *TP53*, *FAT3*, *PDE4DIP*, *KMT2C*, *FAT1*, and *MYO18A* were the most commonly mutated genes, which were distinct from the previous studies (Ling et al., 2018; Gao et al., 2019; Wang et al., 2019; Yang et al., 2023), recognizing that *TP53*, *TERT*, and *CTNNB1* were the most frequently mutated in Chinese HCC. It was implied that *FAT3*, *PDE4DIP*, *KMT2C*, *FAT1*, and *MYO18A* might be novel, potential, and prevalent driver genes frequently stimulating the development and progression of HCC in Chinese patients. Strikingly, our study proved that the aberrant regulation of *FAT3*, *KMT2C*, and *FAT1* was markedly associated with the proliferation and migration of HCC-related cell lines. As reviewed, *FAT1* and *FAT3*, as the components of FAT family genes, could regulate the FAT-Hippo signaling pathway, and remarkably, FAT family gene mutations had been thought to be closely associated with tumor progression (Katoh, 2012). *PDE4DIP*, encoding a protein known as myomegalin, mainly participated in the microtubule dynamics and could regulate the cAMP/PKA signaling pathway (Bouguenina et al., 2017). Impressively, circulating tumor cell DNA (ctDNA) sequencing analysis in a Chinese HCC cohort reported that *PDE4DIP* was the most commonly mutated, followed by *SYNE1* and *KMT2C* (Ding et al., 2022); however, the functional role of PDE4DIP in the development and/or progression of HCC remains to be further elucidated. *KMT2C*, a chromatin remodeling gene encoding a kind of histone methyltransferase, was recently found to be frequently mutated in Taiwanese HCC (Chang et al., 2023) and Chinese HCC (Wang et al., 2022). *MYO18A*, encoding an unusual myosin, had never been reported to be frequently mutated in HCC, though it had been identified as being implicated as a cancer driver (Buschman and Field, 2018), which was also validated by the cell lines in the present study. When focusing on genomic alterations by functional significance, as expected, Hippo signaling pathway-related alterations were prevalent in the Chinese HCC cohort. Altogether, this study suggested several novel insights for the pathogenesis and progression of HCC. Additionally, the alteration frequency of *CTNNB1* was only 14.66% in our HCC cohort, markedly lower than that (28%) in the Western HCC cohort. *CTNNB1* mutations and/or activation of the Wnt signaling pathway could downregulate *CCL5* expression and impair dendritic cell recruitment, resulting in the stimulation of immune escape and resistance to immune checkpoint inhibitors in a mouse model of HCC (Ruiz de Galarreta et al., 2019). It could be inferred that a large number of Chinese HCC patients seemed sensitive to immunotherapeutic interventions.

Similarly, for ICC patients, it was identified in our cohort that *TP53*, *FAT1*, *FAT3*, and *PDE4DIP* were frequently mutated, and the prevalent alterations were enriched in the Hippo, Notch, and TP53 signaling pathways. In addition, in a Western ICC cohort by the MSKCC center, it was shown that *IDH1*, *BAP1*, and *TP53* genes were prevalently altered (Lowery et al., 2018). Perhaps genomic instability in ICC could be associated with genetic backgrounds. However, it should be emphasized that a larger ICC cohort containing Chinese and Western patients was needed to deeply investigate whether ICC patients with distinct genetic backgrounds had distinct genomic characterizations, and moreover, the etiology of HCC should also be taken into consideration in future explorations. Recently, a biliary tract cancer cohort study involving 51 Chinese ICC patients demonstrated that the most frequently mutated genes included *TP53* (57%), *KRAS* (35%), *ARID1A* (16%), and *PIK3CA* (16%), and the most prevalently altered signaling pathways were RTK/RAS (64.7%), TP53 (60.8%), and PI3K (33.3%) (Liu et al., 2022), which was inconsistent, either, compared with genomic characterizations in our cohort. As described before, this discrepancy could be caused by the limited patient number, but from another perspective, *FAT1*, *FAT3*, and *PDE4DIP* might be newly identified driver genes for the pathogenesis of ICC. As discussed before, *FAT1*, *FAT3*, and the associated Hippo signaling pathway play a pivotal role in organogenesis and hemostasis and could regulate the proliferation and apoptosis of liver cells. The Hippo signaling pathway has been recognized to be tightly correlated with the development, progression, and even recurrence of ICC (Mranda et al., 2022). Of note, it was further found in our cohort that *KRAS* (18.18%) and *BAP1* (13.64%) alterations were particularly more frequent among ICC patients than those among HCC patients, implying that KRAS-targeted anti-cancer strategies (Xu et al., 2019) could be hopefully used for *KARS*-mutated ICC patients. Based on the functional role of BAP1 in DNA damage repair, PARP inhibitors and platinum-based chemotherapeutic agents (Louie and Kurzrock, 2020) have the potential to treat *BAP1*-mutated ICC tumors. Moreover, *BAP1*-mutated mesothelioma tumors have a higher level of sensitivity to immunotherapies (Ladanyi et al., 2019), indicating that ICC patients with altered *BAP1* could make a significant response to immunotherapy, but this needed further verification.

Recently, it was found that the downregulation of low-density lipoprotein receptor (*LDLR*) caused increased cholesterol synthesis by the MEK/ERK pathway, leading to the progression and metastasis of HCC, while simvastatin inhibiting cholesterol synthesis could attenuate the progression of HCC under lower LDLR (Chen et al., 2021). Thus, simvastatin could be an effective therapeutic strategy for *LDLR*-altered HCC tumors to prevent VI, but it needed further experimental and clinical validation. Moreover, this is the first time we discovered that *MSH2* mutations were significantly correlated with VIs in HCC. *MSH2*, as a member of the mismatch repair family, is important in maintaining genomic stability, and undoubtedly, *MSH2* mutations could promote the initiation and progression of HCC (He et al., 2022). *KDM5D* was one of the chromatin remodeling genes often regarded as late-occurring tumor suppressors (Kaya et al., 2022); expectedly, this study revealed that its alterations might be highly associated with the progression and VIs in HCC. Notably, mutated *PDE3A* was first identified in the present study to be highly correlated with VIs of HCC. *FOXO1*, encoding a transcription factor, was a common suppressor gene in HCC (Cui et al., 2023), and FOXO1 could regulate the immune response by affecting cytokine expression, immune cell migration, and cellular senescence in macrophages and T cells (Malik et al., 2017; Yang et al., 2018; Delpoux et al., 2021). Consequently, *FOXO1* mutations could cause the dysfunction of immunological modulation, eventually prompting the VI of HCC. Overall, the present study provided an in-depth investigation of the association of genomic characterizations with VIs, pinpointing new possibilities for novel treatment strategies to suppress the invasion and progression of HCC.

In 1994, A. Torii et al. first proposed that tumor localization was a contributory factor in the prognosis of HCC, and HCC patients whose tumors were confined to the left hepatic lobe had a trend toward better prognosis than patients whose tumors were located strictly in the right hepatic lobe (Torii et al., 1994). Strikingly, a similar outcome was obtained in our cohort. In the present study, we compared the genomic differences between the left hepatic lobe and right hepatic lobe in HCC for the first time; we found that *SBNO2* and *IGF1R* were prevalently altered in the right and left hepatic lobes of HCC, respectively. *SBNO2*, namely, strawberry notch homolog 2, is involved in several immunological processes, including cellular response to interleukin-6 (IL-6) and macrophage activation (El et al., 2007). Yet, the function of *SBNO2* in HCC remained unknown, and whether *SBNO2* mutations could affect the development and/or progression of (right hepatic lobe) HCC needed to be further verified. IGF1R, as a ubiquitous receptor tyrosine kinase, could mediate the progression and metastasis of multiple malignancies, and IGF1R inhibitor linsitinib plus PTK2 inhibitor defactinib significantly restrained BACH1-mediated HCC growth and metastasis (Xie et al., 2022). The Notch signaling pathway was pro-tumorigenic, especially in HCC, and the aberrant Notch signaling pathway was closely associated with carcinogenesis, progression, invasion, and even metastasis in HCC (Huang et al., 2019). Altogether, the potential distinct pathogenic mechanisms between the left and right hepatic lobes of HCC indicate that the location of onset should be taken into consideration when developing therapeutic regimens in clinical practice. Comprehensive genomic characterizations revealed that SBNO2 and the associated Notch signaling pathway might be suitable therapeutic targets in the right hepatic lobe of HCC, while the inhibitor linsitinib targeting IGF1R could be effective for the left hepatic lobe of HCC.

Except for *RB1* and *NOTCH3*, altered *SYNE1*, *MGA*, and *ZFHX3* were first discovered to be closely associated with the OS of HCC. It was consistent with previous research that *RB1* mutations were significantly correlated with the shorter OS in Asian HCC patients (Duan et al., 2021). In addition, it has been reported that the aberrant regulation of *NOTCH3* predicted inferior survival for HCC patients (Hu et al., 2013), and targeting NOTCH3 could become a novel and promising strategy for HCC treatment (Giovannini et al., 2017). In addition, RFS is also an important parameter influencing the clinical outcome of HCC. Despite hepatic resection and adjuvant chemotherapy or combination immunotherapy, the recurrence rate of HCC exceeded expectations. Tumor recurrence and metastasis have become the primary causes constraining the long-term survival of HCC after surgical resection. Moreover, it should be noted that a prospective phase Ⅲ STORM trial did not achieve its primary endpoint of improving the RFS when comparing sorafenib with placebo as an adjuvant therapeutic strategy (Pinyol et al., 2019b), though some retrospective studies exhibited that sorafenib treatment after resection could effectively reduce the rate of recurrence and metastasis. Currently, adjuvant systemic anti-cancer therapeutic strategies after resection, such as targeted therapy, adjuvant chemotherapy, and (combination) immunotherapy, remain actively explored. In the present study, genomic characterizations associated with HCC recurrence in Chinese patients were deeply investigated, which demonstrated that *LATS1* alterations as an independent risk factor were significantly associated with HCC recurrence. LATS1 kinase, as a core component of the Hippo signaling pathway, has been shown to exert suppressive activities on tumors (Furth and Aylon, 2017), and it was found that LMO3 could promote the invasion and progression of HCC by interacting with LATS1 and suppressing Hippo signaling (Cheng et al., 2018). Therefore, adjuvant therapeutic strategies targeting LATS1 and/or Hippo signaling pathways might be beneficial in reducing the recurrence rate and improving the clinical outcome of HCC.

In recent years, systemic therapies have been widely proposed for the treatment of HCC, especially for patients in advanced stages. Sorafenib was the first systemic therapy approved for advanced-stage HCC, and it significantly improved the median OS from 7.9 months to 10.7 months (Llovet et al., 2008). In addition, lenvatinib, regorafenib, and cabozantinib have also been proved to improve clinical outcomes, though the median OS remained at approximately 12 months (Llovet et al., 2018). The IMbrave150 study, a global, multicenter, open-label, phase 3 randomized trial (Finn et al., 2020), found that atezolizumab plus bevacizumab-treated HCC patients had a median OS of 19.2 months; in comparison, sorafenib-treated HCC patients had a median OS of 13.4 months (Ducreux et al., 2021). Of the 194 Chinese patients enrolled in the IMbrave150 study, 133 received atezolizumab plus bevacizumab and 61 received sorafenib. Of note, identical improvement was also obtained: anti-PD-L1 plus anti-angiogenesis-treated patients had a median OS over 24 months, but sorafenib-treated patients had a median OS of only 11.4 months (Qin et al., 2021). Nowadays, anti-PD-(L)1 plus bevacizumab has become the first-line systemic treatment for HCC (Llovet et al., 2022); however, it should be emphasized that there are still a large number of HCC patients who do not derive substantial survival benefits. Thus, it was urgently needed to discover novel biomarkers that could predict response or resistance to immunotherapy in HCC. First of all, the present study investigated the predictive value of TMB and identified that the increased TMB level represented a higher objective response rate, improved OS, and significantly better PFS. Similarly, as previously reported, lower TMB levels might limit the role of TMB as a predictor of response to (combination) immunotherapy in HCC (Wong et al., 2021). In addition, this study identified several novel genetic predictors, comprising *KMT2D*, *PTPRZ1*, and cell cycle signaling-related mutations, in response to anti-PD-1 plus bevacizumab in HCC. Notably, *KMT2D* alterations could restrain the response to anti-PD-1 plus bevacizumab, eventually leading to an inferior PFS. In contrast, a significantly higher objective response rate and a slightly improved PFS occurred among patients with altered *PTPRZ1*. As previously reported, PTPRZ1 could activate the β-catenin pathway (Xia et al., 2019), leading to an immune-suppressive microenvironment. Thus, it was inferred that *PTPRZ1* alterations might result in an immune-promoting microenvironment. In addition, enhanced cell cycle activity in cancer cells could suppress anti-tumor immunity, and pharmacological inhibition of the cell cycle can promote anti-tumor immunity and improve the response to immunotherapy (Li and Stanger, 2020). As found in the present study, cell cycle signaling-related alterations could be of great value in predicting the response to anti-PD-1 plus bevacizumab in HCC. More impressively, the characterizations of TMB-high, wild-type *KMT2D*, altered *PTPRZ1*, and altered cell cycle signaling were positively associated with the PD-1 expression in this study, and as discussed previously (Zhu et al., 2022), PD-(L)1 expression was closely associated with the improved survival and response of immunotherapy among patients with advanced HCC.

In the present study, there were still some limitations. First, the number of HCC patients enrolled in the clinical trial of sintilimab (anti-PD-1) plus bevacizumab treatment was a little small; thus, a larger patient cohort was necessary for future validation. Second, we investigated the association between genetic alterations and immunotherapy responses and PFS in HCC, but their functional role should be further elucidated. Third, the underlying mechanisms were still unknown, and experimental designs were necessary for the verification of the cell lines and/or mouse model.

## 5 Conclusion

Comprehensive genomic profiling of Chinese patients with liver cancer displayed differential pathogenic mechanisms between HCC and ICC, and comparison analysis further exhibited the distinct genomic characterizations between patients with different genetic backgrounds. Moreover, several altered genes were first identified to be closely associated with the progression, invasion, and recurrence of HCC, and some of them could predict the OS and RFS as independent prognostic factors. More impressively, three novel genetic predictors were identified to be markedly associated with the response to anti-PD-1 plus bevacizumab, OS, and PFS in HCC. Overall, this study provided novel insights into the development and progression of liver cancer, which would help develop more therapeutic strategies and promote the clinical management of liver cancer.

## Data Availability

The data presented in the study are deposited in the China National Center for Bioinformation, Genome Sequence Archive for Human, accession number: HRA005058 (available at: https://ngdc.cncb.ac.cn/search/specific?db=hra&q=HRA005058).
