# Supplementary figures and images for: Novel genetic alterations in liver cancer distinguish distinct clinical outcomes and combination immunotherapy responses
Source: Front Pharmacol. 2024 Jun 14;15:1416295. doi: 10.3389/fphar.2024.1416295 (PMC11211383; doi:10.3389/fphar.2024.1416295)

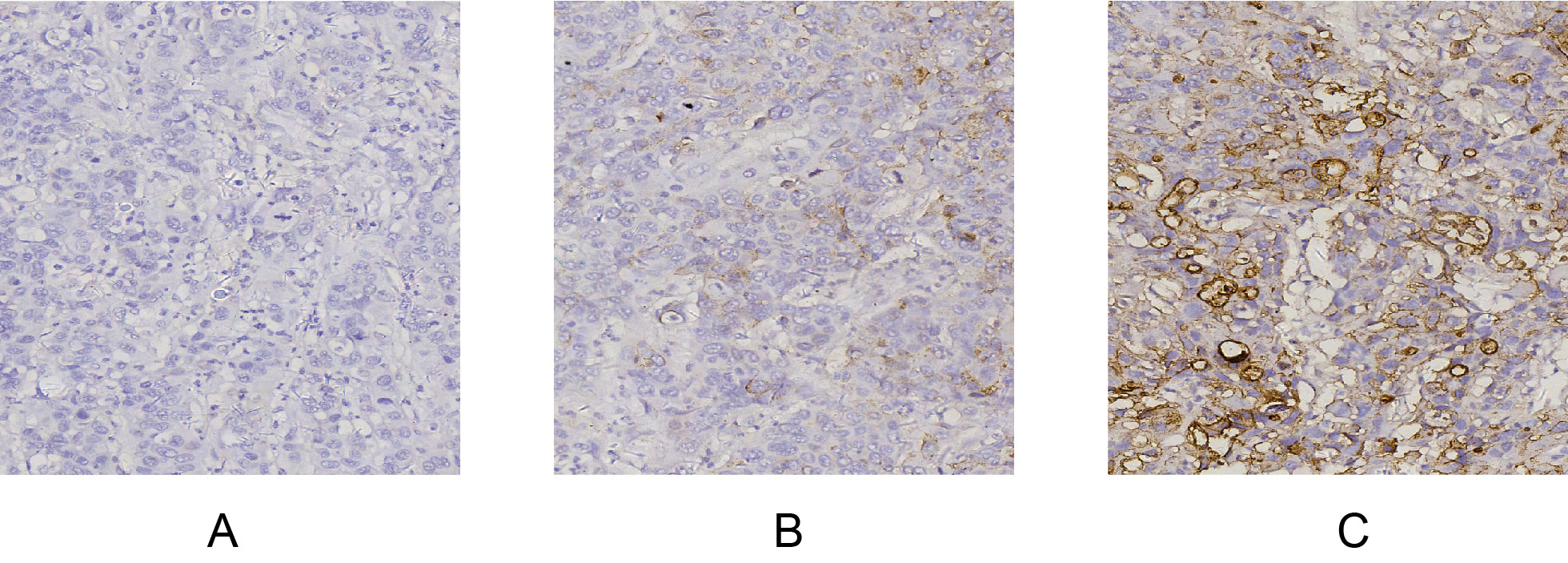

Supplement: Supplementary file 1 [file Image3.JPEG]

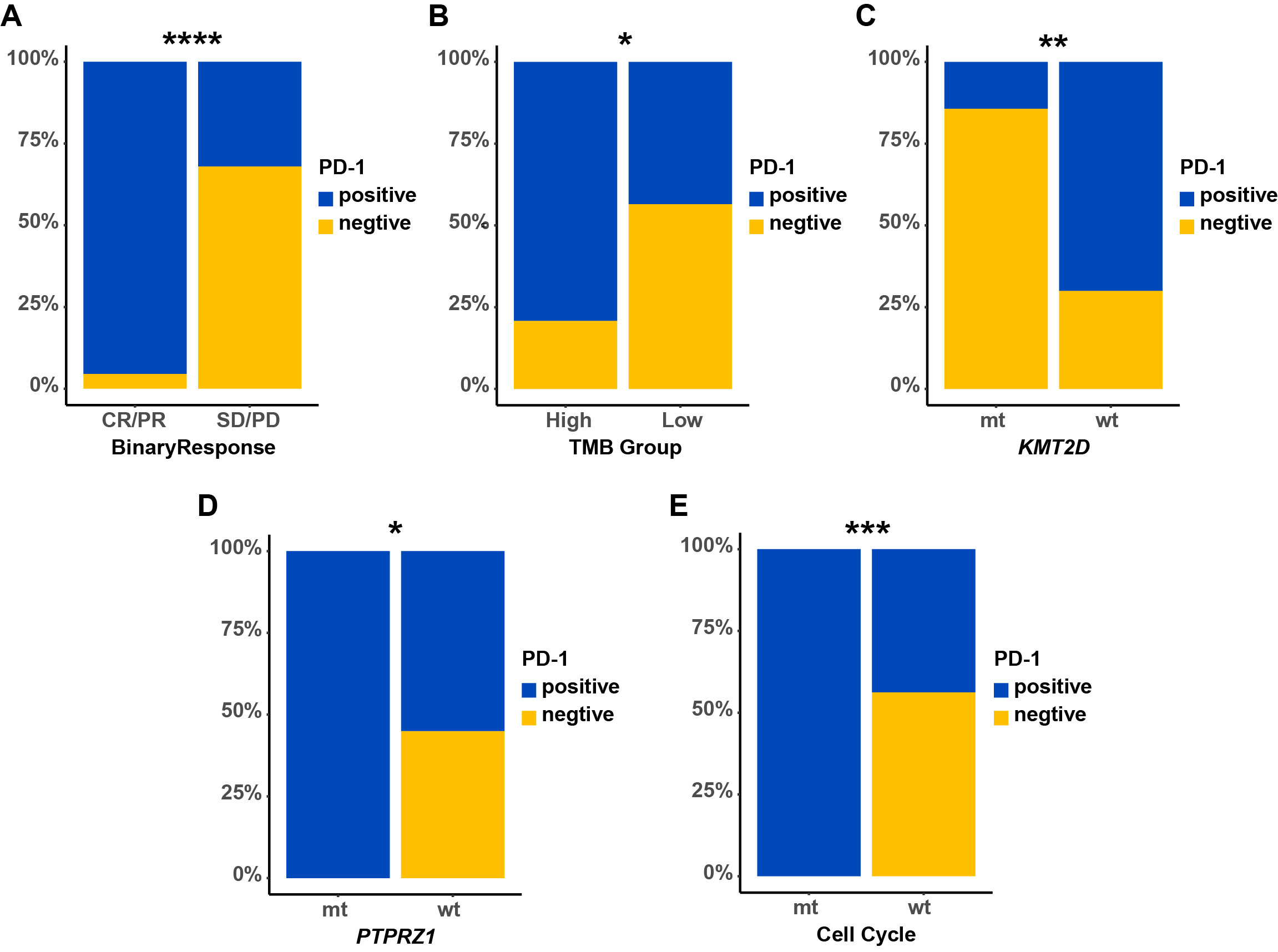

Supplement: Supplementary file 2 [file Image4.JPEG]

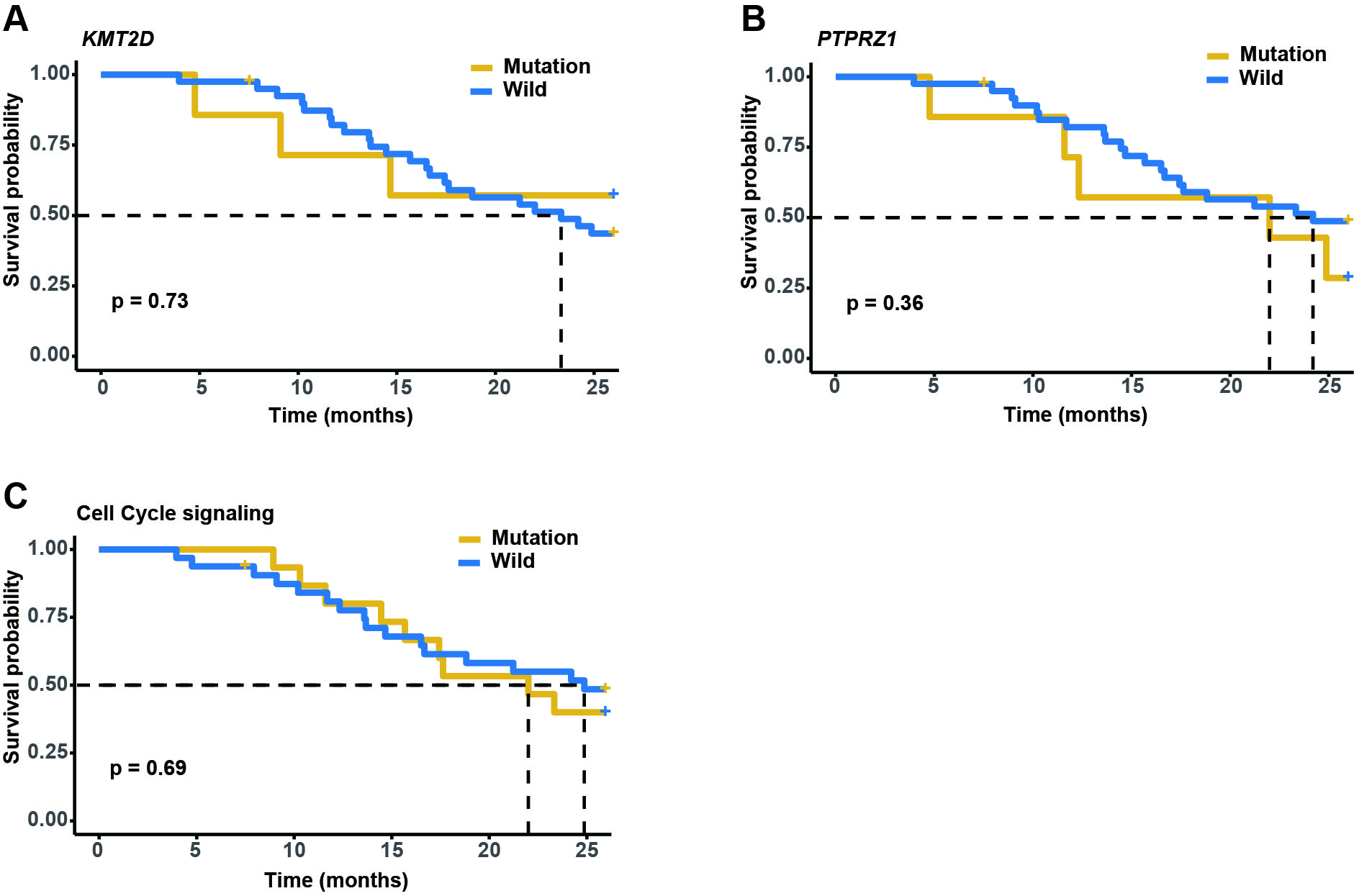

Supplement: Supplementary file 3 [file Image2.JPEG]

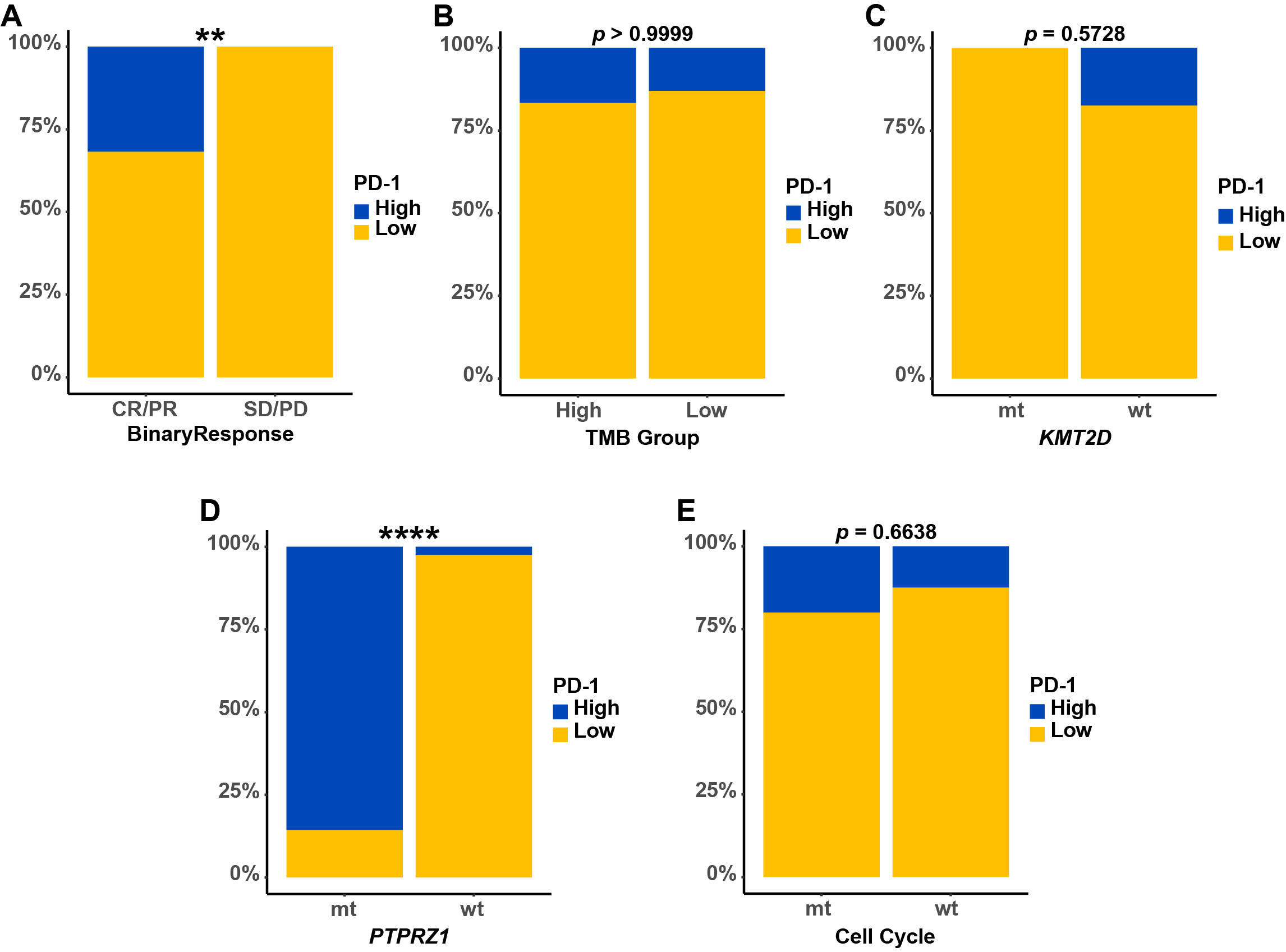

Supplement: Supplementary file 4 [file Image5.JPEG]

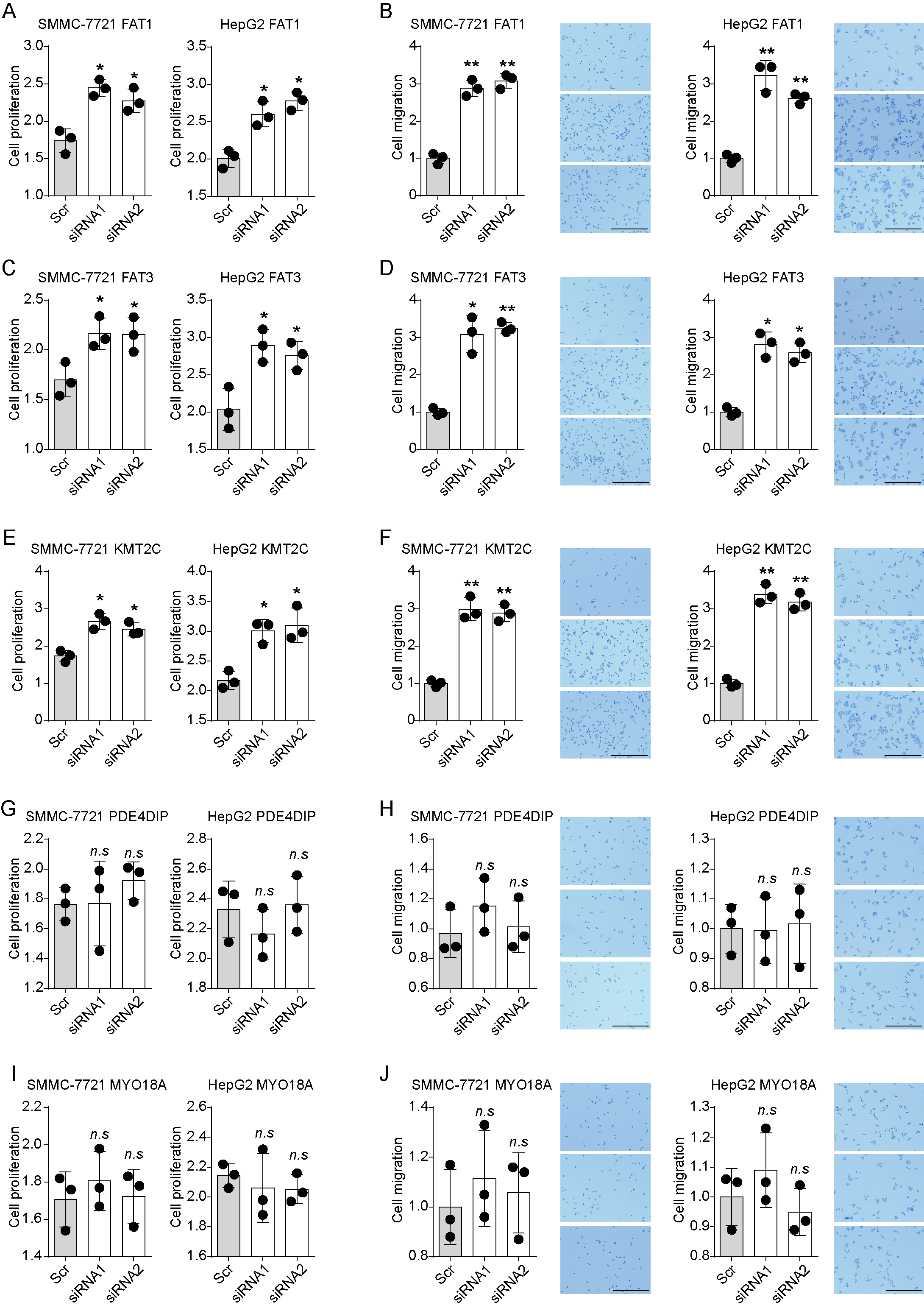

Supplement: Supplementary file 5 [file Image1.TIF]
